# Large and accessible conductivity of charged domain walls in lithium niobate

**DOI:** 10.1038/s41598-017-09703-2

**Published:** 2017-08-29

**Authors:** Christoph S. Werner, Simon J. Herr, Karsten Buse, Boris Sturman, Elisabeth Soergel, Cina Razzaghi, Ingo Breunig

**Affiliations:** 1 Department of Microsystems Engineering - IMTEK, University of Freiburg, Georges-Köhler-Allee 102, 79110 Freiburg, Germany; 2 Fraunhofer Institute for Physical Measurement Techniques IPM, Heidenhofstraße 8, 79110 Freiburg, Germany; 3 Institute for Automation and Electrometry of Russian Academy of Science, 630090 Novosibirsk, Russia; 4 Institute of Physics, University of Bonn, Wegelerstraße 8, 53115 Bonn, Germany

## Abstract

Ferroelectric domain walls are interfaces between areas of a material that exhibits different directions of spontaneous polarization. The properties of domain walls can be very different from those of the undisturbed material. Metallic-like conductivity of charged domain walls (CDWs) in nominally insulating ferroelectrics was predicted in 1973 and detected recently. This important effect is still in its infancy: The electric currents are still smaller than expected, the access to the conductivity at CDWs is hampered by contact barriers, and stability is low because of sophisticated domain structures or proximity of the Curie point. Here, we report on large, accessible, and stable conductivity at CDWs in lithium niobate (LN) crystals – a vital material for photonics. Our results mark a breakthrough: Increase of conductivity at CDWs by more than 13 orders of magnitude compared to that of the bulk, access to the effect via ohmic and diode-like contacts, and high stability for temperatures *T* ≤ 70 °C are demonstrated. A promising and now realistic prospect is to combine CDW functionalities with linear and nonlinear optical phenomena. Our findings allow new generations of adaptive-optical elements, of electrically controlled integrated-optical chips for quantum photonics, and of advanced LN-semiconductor hybrid optoelectronic devices.

## Introduction

The ferroelectric state can be characterized by the spatial distribution of the vector of spontaneous polarization ***P***_S_; within a ferroelectric domain ***P***_S_ is constant. Ferroelectric domain walls (neutral or charged) separate regions with different polarizations. Owing to the local breaking of spatial symmetry, they can exhibit properties entirely different from those of the undisturbed material^1–4^. It is found, in particular, that neutral ferroelectric domain walls exhibit pronounced conductive properties^5–8^. Nowadays the domain walls can be created, displaced, deleted, and recreated again^2, 9, 10^. They are expected to serve as functional active elements of future nano-electronics^2–4, 11^.

**Figure 1:**
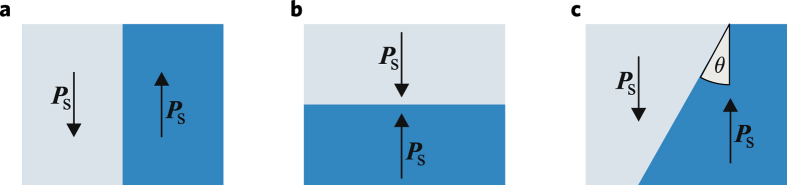
Three elementary types of domain walls with opposite directions of ***P***_S_: a neutral wall (**a**), a 180**°** head-to-head CDW (**b**), and a *θ*-inclined head-to-head CDW (**c**).

Often the ferroelectric domain walls are neutral, as illustrated by Fig. 1a, so that the polarization difference Δ***P***_S_ is parallel to the wall plane and the bound polarization charge is zero. Otherwise, the walls are charged and the bound surface charge is almost completely compensated by free charges of the opposite sign (electrons, holes, or ions), thereby providing the ferroelectric stability^12, 13^. Figures 1b and c illustrate two types of charged domain walls (CDWs) with opposite directions of the spontaneous polarization. For these head-to-head walls, the bound surface charge is + 2*P*_S_ and + 2*P*_S_ sin*θ*, respectively, where *θ* is the inclination angle, and the compensating negative surface charge is almost opposite in value.

It was predicted in 1973 that CDWs in nominally insulating ferroelectrics possess metallic-like conductivity owing to the movement of the compensating electrons or holes along the wall^14^. Difficulties with creation, stability, and control of CDWs hampered detection of this phenomenon. Nevertheless, CDWs and signs of their elevated conductivity were occasionally documented in the past, see Refs. 12, 13 and references therein. During the last years, pronounced DC conductivity at CDWs was detected in many materials including PbZr_0.2_Ti_0.8_O_3_ films^15^, BaTiO_3_^12, 16^, BiFeO_3_^17^, ErMnO_3_^18^, hexagonal h-HoMnO_3_^19^, and (Ca,Sr)_3_Ti_2_O_7_ crystals^20^. So far, the most advanced data for CDWs has been presented for BaTiO_3_ single crystals: An enhancement factor of ∼10^9^ as compared to the bulk conductivity was obtained for 45° head-to-head walls, and signs of metallic-like behaviour were indicated^12^.

High-resolution piezoresponse force microscopy (PFM) and conductive atomic force microscopy (c-AFM) are extensively in use to investigate the domain wall conductivity^3^. The presence of substantial contact barriers leading to non-ohmic behaviour and an insufficient temporal stability hamper often the progress in the field^3, 12, 13^. The tip-related c-AFM measurements are often qualitative, so that the absolute values of the domain-wall conductivity remain usually unknown. Typical DC currents lie in the pA range. Direct observations of ionic displacements inside CDWs are rare^21, 22^. They are sufficient to estimate the structural width to be ~1 nm, but insufficient to judge about the charge compensation and transport mechanisms. The latter are the subject of modelling^23–25^. It is expected thus that the conductive width *w* of charged domain walls is about 10 nm, exceeding the width of neutral walls by roughly one order of magnitude.

Our choice of lithium niobate (LN) crystals for the studies of domain-wall conductivity has strong grounds: This robust wide-band-gap ferroelectric (*E*_g_ ≈ 4 eV) is extensively applied in optics^26–28^. It has a Curie temperature as high as *T*_C_ ≈ 1200 °C, large values of the spontaneous polarization near room temperature, *P*_S_ ≈ 70 µC/cm^2^, and only two allowed opposite values of ***P***_S_^29^. Field-assisted domain engineering of LN is a well-developed area because of the relevance of quasi-phase-matching in nonlinear optics^26, 30, 31^. It is known that engineered domain structures can possess in LN CDWs of different configurations^32–34^. The dark conductivity of LN crystals is very low^29^; at room temperature it is well below 10^−15^ (Ω cm)^−1^. This corresponds to a very slow (with relaxation times of months or years) migration of numerous ions^35^, so that the electronic compensation of CDWs via the band bending^13, 23–25^ looks the most natural on a realistic time scale.

The previous studies of the electrical properties of CDWs in LN crystals have shown only a transient conductivity during the polarization switching^36^ and a DC conductivity with ohmic and diode-like behaviour in the presence of super-band-gap illumination^37^. Furthermore, AC transport along CDWs, bypassing contact barriers through capacitive coupling, was reported^38^. Very recently, after completion of this manuscript^39^, diode-like *J*-*U* characteristics in LN thin-films have been shown^40^, and large DC currents along CDWs without illumination were demonstrated in bulk crystals^41^. We present a new method for the fabrication of charged domain walls, measure their specific surface conductivity and reveal the prerequisites to obtain ohmic contacts. By using bulk crystals, we can distinguish the influence of the domain wall from the surface effects. This allows us to provide an equivalent circuit diagram, which shows the ohmic contribution of the bulk material as well as the position of the ohmic and diode-like interfaces. Additionally, a systematic analysis of the temperature influence on the conductivity and decay times indicates the types of charge transport and compensation mechanisms. This also allows a prediction of the long-term stability.

## Results

### Experimental implementation

**Figure 2:**
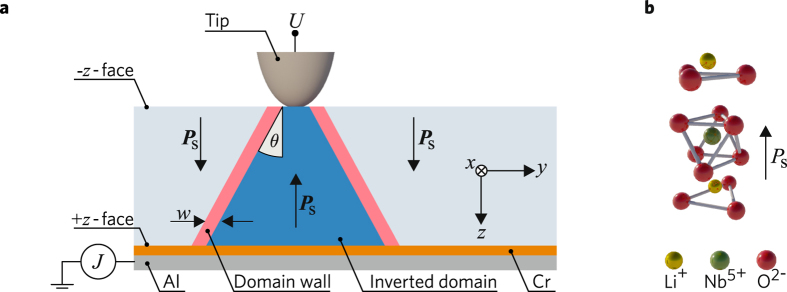
(**a**) Schematic of the experimental setup and of an inverted domain. The inclination angle *θ* and the width *w* of the charged domain wall are sketched exaggeratedly. The coordinate system (*x*, *y*, *z*) coincides with the crystallographic axes of lithium niobate. (**b**) Atomic structure of lithium niobate with the corresponding polarization vector ***P***_S_.

The results presented below were achieved with 300-μm-thick *z*-cut samples of congruent LN doped with 5 mol. % MgO. In order to produce mm-long charged domain walls in originally single-domain crystals, we extended calligraphic domain writing^30^ with an active current stabilisation. Our experimental setup allowing this and subsequent conductivity measurements is presented schematically in Fig. 2a. The bottom + *z*-face of the sample is coated with a 0.5-μm-thick Cr electrode and then glued with conductive paste to a heatable Al mount. The temperature *T* can be controlled on the 0.1 °C level within the range (20–150) °C.

For domain inversion we used homemade tungsten-carbide tips with μm-sized radii. In the subsequent measurements, we employed not only the μm-sized tips, but also a commercial AFM with c-AFM and PFM probes of about 100 nm radii in order to verify the results with an established method. Furthermore, this provides a higher spatial resolution. In ambient air, a water meniscus blurs the image in motion direction of the tip^42^.

During domain-inversion, the tip moves in the *x*-direction producing a domain line of approximately 10 μm width and a few mm length. The applied voltage *U* is controlled by an electronic feedback-loop such that the poling current stays constant at about 30 nA, while the crystal is kept at room temperature. Subsequent selective etching of the sample shows that the domain size on the + *z*-face noticeably and reproducibly exceeds the size on the -*z*-face. Thus the generated domain walls are inclined and positively charged, see also Fig. 2a. The inclination angle for CDWs can be estimated as *θ* ≈ 1°. A similar feature of domain walls in LN crystals is mentioned in Ref. 37 for various generated domain structures. Poling in the *y-*direction gives similar domain lines. Remarkably, the total charge that passes the sample during the domain-inversion procedure exceeds the net polarization charge by orders of magnitude. This is already an indication of CDW conductivity.

### Localization of the conductivity

**Figure 3:**
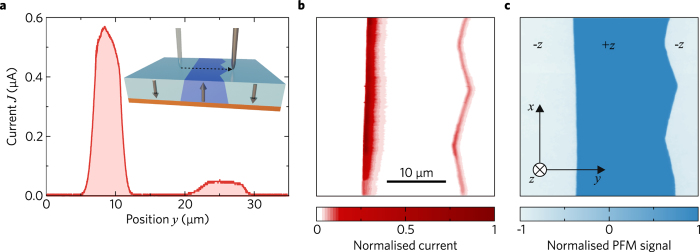
(**a**) Current *J* versus tip position *y* when scanning across the recorded domain line with a voltage of *U = *50 V applied to the tip. (**b**) Spatial distribution *J*(*x*,*y*) obtained with a c-AFM probe. (**c**) Corresponding PFM image identifying the ± *z* domains.

In order to investigate the origin of the high poling-current, we move with a speed of 10 µm/s an electrically-biased μm-sized virgin tip across the domain line in the *y*-direction, as shown in the inset of Fig. 3a. To avoid any further field-induced changes of the domain structure, the bias-voltage *U* is set to 50 V, i.e. the applied field stays far below the coercive field (*E*_c_ ≈ 3 kV/mm). The current *J*(*y*) is nonzero only while crossing the two domain walls; this proves that CDWs are responsible for the conduction. The value *J*_max_ is as high as 0.55 µA. The peak currents of the two adjacent domain walls show a systematic asymmetry. Employment of a c-AFM probe has allowed us to map the spatial current distribution in the *x*-*y* plane, see Fig. 3b. Also here, we find a highly conductive domain wall and a less conductive one. The first appears as a straight line whereas the second has a zigzag shape. The corresponding PFM image, see Fig. 3c, confirms that the conductivity is localised at the position of the domain walls. All investigated zigzag-shaped domain walls exhibit a smaller conductivity than their straight counterparts.

In near-stoichiometric 1.2-mol.%-MgO-doped LN we found the same phenomena, i.e. also in this material currents of similar magnitude can be observed.

### Current-voltage characteristics

**Figure 4:**
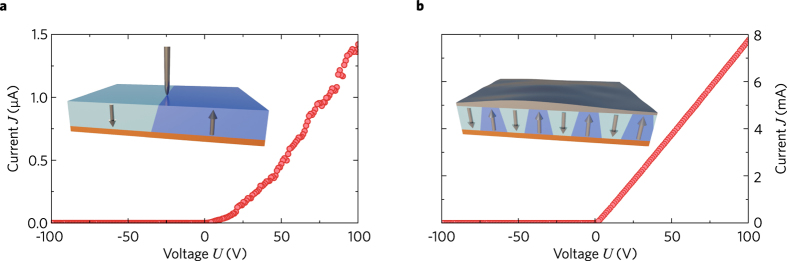
(**a**) Dependence *J*(*U*) at the point of maximum of *J*(*y*) for a single domain line. (**b**) *J*(*U*) for an array of 732 domain lines covered with a conductive silver-paste top electrode.

Next, we positioned our μm-sized tip at the point of maximum of *J*(*y*) and measured the current-voltage (*J*-*U*) characteristic. The corresponding result is presented in Fig. 4a.

A pronounced diode-like dependence is clearly seen. For *U* < 0 the current is practically absent, while for positive voltages we have an almost linear *J*-*U* dependence. This Schottky-like *J*-*U* characteristic is typical for CDW experiments and implies the existence of a contact barrier. The linear increase of the current with respect to voltage for *U* > 0 indicates a pronounced ohmic contribution to the conductivity, i.e. a series connection of a single diode and a resistor. The *J*(*U*) measurement has then been advanced further: Instead of a single line we have fabricated an array of 732 parallel 1-mm-long domain lines. After that, the top face was covered with conductive silver paste contacting all CDWs in parallel. This configuration shows also a diode-like *J-U* characteristic, see Fig. 4b. However, the values of the current for *U* > 0 approach 0.01 A and the signal-to-noise ratio is significantly higher.

**Figure 5:**
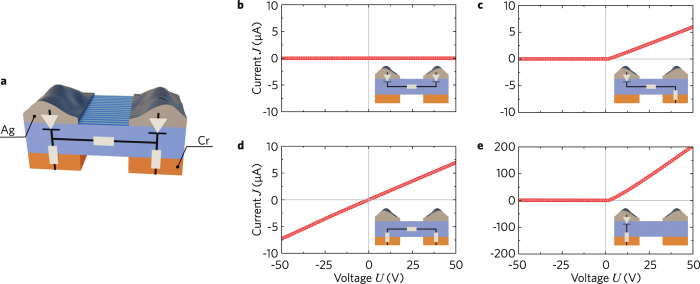
Current measurements using four electrodes. (**a**) Geometry and equivalent electric scheme. (**b**) Top-Top connection: zero current. (**c**) Top-left-Bottom-right connection: semi-ohmic diode-like behaviour. (**d**) Bottom-Bottom connection: fully ohmic behaviour. (**e**) Top-left-Bottom-left connection: diode-like behaviour.

In the previous paragraph, we revealed an asymmetry of the *J-U* characteristics. This is a strong indication that the contact barriers on the top and bottom surfaces are different. This effect has been studied further: We have fabricated an array of sixteen 13-mm-long domain lines. Then, the central part of the bottom Cr electrode has been etched away, and two spatially separated droplets of conductive paste are used as two large-area top electrodes, see Fig. 5a. Zero current occurs when the voltage is applied between the two top electrodes, see Fig. 5b. This shows unambiguously that the top electrodes act as opposing diodes. Applying the voltage between the two bottom electrodes, Fig. 5d, we obtain a fully ohmic behaviour. This indicates that the bottom interfaces between the Cr electrodes and the crystal form ohmic contacts. Both crosswise connections – applying the voltage between the left-top and right-bottom or between the right-top and left-bottom electrodes – return us to the known diode-like behaviour, see Fig. 5c and e. An equivalent circuit diagram of our four-electrode configuration is presented in Fig. 5a.

The ohmic interface is lost, however, when the original Cr electrode is removed and replaced by a new one. The latter leads to a diode-like interface. Thus, the Cr-crystal interface, forming an ohmic contact during the poling process, is crucial for accessing the conductivity of the domain walls.

### Impact of temperature on the conductivity

**Figure 6:**
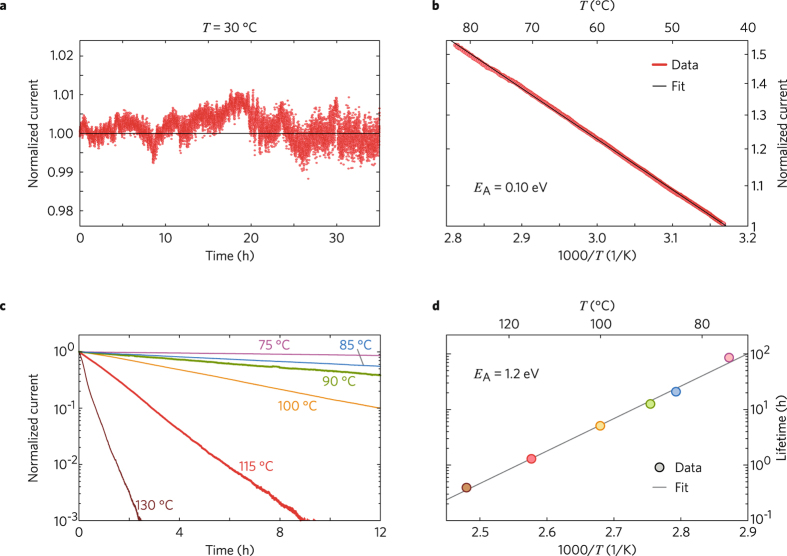
(**a**) Temporal dependence *J*(*t*) at 30 °C. (**b**) Arrhenius plot of steady-state value of *J*(*T*) for the approximate temperature range (40–80) °C. (**c**) Decay of *J* for *T* = 75, 85, 90, 100, 115, and 130 °C. (**d**) Temperature dependence of conductivity decay in the high-temperature range.

The temperature dependence of the CDW conductivity is important for a deeper understanding of the charge transport mechanism. For these studies, we used a sample with thirty 1-mm-long domain lines and a silver-paste top electrode. For *T* ≤ 70 °C, the conductivity shows no signs of temporal degradation. Figure 6a shows an exemplary measurement of *J*(*t*) during 35 hours at *T* = 30 °C. The current does not change within the measurement accuracy. It fluctuates by only 1 % around an average value. Compared with experiments on BaTiO_3_ and BiFeO_3_, the fluctuations are considerably smaller^2, 6, 12^.

The high temporal stability allows us to measure *J*(*T*) for *T* ≤ 70 °C. It obeys an Arrhenius law with the activation energy *E*_A_ = (0.10 ± 0.01) eV, see Fig. 6b. This is the signature of conductivity by electron hopping^43^. For *T* > 70 °C, the current decays exponentially in time, *J*(*t*)/*J*(0) = exp(−*t*/*τ*), see Fig. 6c. The higher *T*, the faster is the decay. Once the conductivity has vanished, it cannot be recovered. Thus, *τ* is the lifetime of the CDW conductivity. It obeys an Arrhenius law with the activation energy *E*_A_ = (1.2 ± 0.1) eV (Fig. 6d). This value strongly indicates that migration of ions is responsible for the conductivity decay at high temperatures. A linear extrapolation to lower *T* predicts that the CDW conductivity lasts for years at room temperature.

### Quantification of the CDW conductivity

The experimental data shown in Fig. 4 allow us to quantify the CDW conductivity. For this purpose, we calculate the total resistance of the system *R* = *U*/*J* under the assumption of ohmic contacts. The problem is two-dimensional. Generally, we have for a single wall *R*_1_ = *ρ*_s_ *F*, where *ρ*_s_ = (*σ w*)^−1^ is the specific surface CDW resistivity having the dimension of Ohm^44^, *σ* is the average conductivity at the wall, *w* is the CDW thickness, and *F* is a dimensionless factor determined by the geometry of the contacts. Let *L*, *a*, and *d* be the domain-line length, the electrode length along the wall, and the sample thickness, respectively.

**Figure 7:**
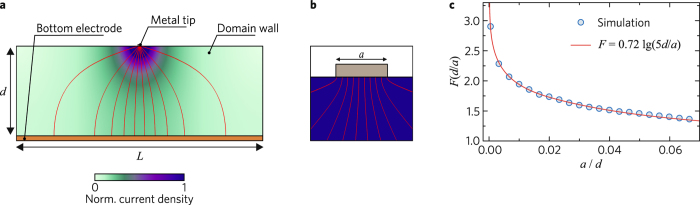
Finite-element simulation for the current density and the resistance in the case of a single domain wall: (**a**) The current density distribution for the wall length *L* = 1 mm, the crystal thickness *d* = 300 µm, and the top electrode length *a* = 10 µm. The current density is color-coded, and the red lines indicate the stream lines. (**b**) Stream lines near the top electrode. (**c**) Dependence of *F* on the ratio *a/d* for *L* = 1 mm.

In the tip-electrode case, the stream lines of the electric current density are expanding in the wall plane with increasing distance from the top electrode, as illustrated by Fig. 7a. The resistance *R* grows when the size *a* of the top-contact area is decreasing. The standard 2D resistivity problem was solved numerically for *d* = 300 µm and different values of *a*, see Fig. 7c. For *L* = 1 mm and *a*/*d* ≪ 1 the factor *F* can be well approximated by the function *F* ≈ 0.72 lg(5 *d*/*a*). The logarithmic dependence on *d*/*a* is weak, so that some uncertainty in our knowledge of *a* is not crucial. Setting *a* = 5 μm, which is not far from the tip diameter, and estimating *R*_1_ = *U*/*J* from Fig. 3a, we obtain *ρ*_s_ ≈ 3.0 × 10^7^ Ω.

For the continuous top electrode we have *a* ≅ *L* ≫ *d*. Here, the stream lines of the current density are parallel to each other and, by analogy with the plane capacitor case, we have *F* ≅ *d*/*L*. For *N* parallel-connected CDWs we have *R*_*N*_ ≈ *ρ*_s_ *d/(N L)*. Setting *L* = 1 mm, *N* = 732 × 2, and estimating *R*_*N*_ = *U*/*J* from Fig. 3b, we obtain *ρ*_s_ ≈ 6.2 × 10^7^ Ω. Thus, two values of *ρ*_s_, estimated for different contacts and geometries, agree well with each other.

Setting *ρ*_s_ = 5 × 10^7^ Ω and *w* = 10 nm, we obtain for the bulk CDW conductivity *σ* ≈ 0.02 (Ω cm)^−1^. This value exceeds the dark conductivity of LN crystals by at least 13 orders of magnitude^45^. Note that our estimate of *σ* is conservative. Switching to the conductive wall thickness *w* ≈ 1 nm, we obtain a one-order-of-magnitude larger value of *σ*.

## Discussion

In addition to detecting the dark DC domain-wall conductivity, that was not accessed in early experiments with LN crystals^36, 37^, we have managed to measure DC currents up to several mA for CDW structures created in this important material. The scale of the CDW conductivity agrees with numbers presented in an independent recent report^41^. We reached full access to the CDW conductivity via diode-like and ohmic contacts. It is evident that the permanent presence of the metal electrode during and after domain inversion is necessary to form an ohmic contact interface. The ohmic access to the CDW has allowed us to reliably determine its specific surface conductivity. Under a conservative estimate, this gives *σ* ≈ 0.02 (Ω cm)^−1^ and at least 13 orders of magnitude enhancement of *σ* as compared to the bulk value. Using the relation *σ* = *e µ*_*e*_ *n* and setting for the electron drift mobility *µ*_e_ ≲ 0.1 cm^2^/Vs, which is true for hopping transport^43^, we obtain for the concentration of compensating electrons the inequality *n* ≳ 1.3 × 10^18^ cm^−3^. It is consistent with the estimate *n* = 2*P*_*S*_ sin *θ*/*e w* ≈ 2 × 10^19^ cm^−3^ for *θ* ≈ 1° and *w* = 10 nm. For *µ*_*e*_ ≈ 0.01 cm^2^/V s, which is a realistic value, we have two close estimates of *n*.

The long-term stability of the currents at temperatures below 70 °C has allowed us to reveal an activation energy of ≈ 0.1 eV. This indicates that the CDW conductivity in LN crystals is based on the hopping transport of electrons rather than on motion of free charges as in metals. The ratio σ/σ_metal_ is about six orders of magnitude large. It comes from the smallness of the electron concentration *n* ≈ 2*θ P*_s_/*ew* compared to *n*_metal_ and also from the smallness of the hopping electron mobility *μ* compared to *μ*_metal_. The latter is not surprising because of non-atomic roughness of slightly inclined CDWs. By increasing *θ* and the smoothness of CDWs one may gain some orders of magnitude in σ.

Changing to a higher temperature range, 75 to 130 °C, we have found a thermally induced decay of the CDW conductivity corresponding to an activation energy of ≈ 1.2 eV. This decay law predicts years of lifetime at room temperature. An exact mechanism of this decay remains unknown. At the same time, a strong correlation with the dark lifetime of refractive-index changes recorded by light in this material, i.e. the photorefractive effect, is remarkable.

Looking forward, it will be relevant to fully elucidate the effect reported here, in particular with the aim to control all parameters: In-depth understanding is needed how the inhomogeneous field employed for the calligraphic poling plus defects that pin the domain walls influence the key outcome, the tilt of the domain walls. However, already now it is clear that this effect can pave the way to novel integrated electronic-optical devices: Calligraphically written charged domain walls can serve in a *z*-cut LN wafer as electrodes inside the bulk to access the electro-optical coefficient *r*_22_, providing electrically controlled local phase modulation. Patterning the wafer and contacting the electrodes with nanometre-thin Ag wires allows the design of freely programmable optical phase plates. Our findings will be useful for integrated reconfigurable quantum-optical devices providing, e.g., correlated photons from electro-optically tunable difference-frequency generation. Flip-bonding a Si chip onto LN samples with tailored conducting domain walls allows one to apply freely designed 2D-structured electrical fields to LN crystals, stimulating the field of LN-semiconductor hybrid circuits.

## Methods

### Sample preparation

All experiments were carried out with commercially available 300-µm-thick z-cut 5-mol.%-MgO-doped LiNbO_3_ crystals (HC Photonics) and with near-stoichiometric 1.3-mol.%-MgO-doped LiNbO_3_ crystals (Oxide Corporation). A chromium layer of approximately 500 nm was sputtered onto the optically polished + *z*-face of the wafer. The optically polished -*z*-face was left uncoated. Subsequently, the wafer was cut into 5 × 15 mm² large chips by means of a wafer-dicing saw. The 15-mm-long edges of the chips were parallel to the crystal *x*-axis.

### Poling and current measurement setup

The setup for calligraphic domain inversion and current measurement comprises two perpendicularly aligned high-precision linear stages (Aerotech ANT95-L) with a computerized numerical control. One stage is equipped with a temperature-controlled aluminium block to heat the crystal. The other stage carries a pivot-mounted cantilever to support the poling tip. A permanent magnet, mounted at the cantilever, and a static coil allow the adjustment of the contact force of the poling tip. Super-fine grain tungsten carbide rods (K-55SF) with 1 mm diameter serve as the starting material to prepare the poling tips. Taper grinding defines the tip geometry, and subsequent polishing with a diamond slurry ensures a tip radius of less than 10 µm (Extended Data Fig. 1).

For domain inversion, the poling tip is connected to a high-voltage source (Trek Model 10/10) while the crystal is glued to the aluminium block using silver conductive paste with the chromium coated side facing towards the aluminium. This ensures good electrical and thermal contact of the chip to the aluminium block. The tip approaches the crystal surface, achieves contact, and then moves along the crystal *x*-axis (Fig. 2b). Simultaneously, a homemade trans-impedance amplifier and a proportional-integral (PI) servo loop control the voltage at the poling tip to ensure a constant poling current of 30 nA. All domain lines were written at room temperature.

For measuring the conductive properties of the domain walls, the poling tip is connected to a high-voltage source with a lower maximum voltage but better signal-to-noise ratio (Trek Model 50/750). A commercial picoamperemeter (Keithley Picoammeter 6485) measures the current through the domain wall at a given tip voltage. The tip can either move perpendicular across the previously written domain lines to obtain a spatial conductivity pattern or can be placed stationary right above a domain wall. Alternatively, conductive silver-paste, simultaneously connecting multiple domain lines at once, acts as a top electrode after domain inversion.

### Piezoelectric force measurements (PFM) and conductive atomic force measurements (c-AFM)

The measurements were carried out with a commercial atomic force microscope (NTEGRA from NT-MDT) equipped with a supplementary external lock-in amplifier (SR 830 from Stanford Research Systems) for the PFM measurements and a low noise current amplifier (DLPCA-200 from FEMTO Messtechnik GmbH) for the c-AFM measurements. The probes used were diamond coated (HA_HR_DCP from NT-MDT), exhibiting a tip radius of about 100 nm. For the PFM measurements, we applied an alternating voltage (*f* = some 10 kHz, *U*_pp_ = 15 V) to the tip, and recorded the in-phase output channel of the lock-in amplifier. For the c-AFM measurements, we applied a DC voltage of 10–100 V to the Cr-bottom electrode, and the current was collected from the tip using the current amplifier. Typical scanning speed was set to few µm/s.

### Additional Information

After submission of the manuscript, the experimental data on large DC currents through CDWs in LN crystals have been published^41^. Our results are consistent with these data and provide additional insights on a variety of properties of the CDW conductivity in this material.

## Electronic supplementary material

Supplementary Information
