# Supplementary material for: Large and accessible conductivity of charged domain walls in lithium niobate
Source: Sci Rep. 2017 Aug 29;7:9862. doi: 10.1038/s41598-017-09703-2 (PMC5575345; doi:10.1038/s41598-017-09703-2)
Supplement: Supplementary file 1 — Supplementary Information [file 41598_2017_9703_MOESM1_ESM.pdf]

# - Supplementary Information -

Large and accessible conductivity of charged domain walls in lithium niobate

Christoph S. Werner[1,*], Simon J. Herr[1,*], Karsten Buse[1,2], Boris Sturman[3], Elisabeth Soergel[4], Cina Razzaghi[4], and Ingo Breunig[1,2]

[1]Department of Microsystems Engineering - IMTEK, University of Freiburg, Georges-Köhler-Allee 102, 79110 Freiburg, Germany

[2]Fraunhofer Institute for Physical Measurement Techniques IPM, Heidenhofstraße 8, 79110 Freiburg, Germany

[3]Institute for Automation and Electrometry of Russian Academy of Science, 630090 Novosibirsk, Russia

[4]Institute of Physics, University of Bonn, Wegelerstraße 8, 53115 Bonn, Germany

[*]These authors contributed equally to this work

Methods

Sample preparation. All experiments were carried out with commercially available 300-µm-thick z-cut 5-mol.%-MgO-doped $LiNbO_3$ crystals (HC Photonics) and with near-stoichiometric 1.3-mol.%-MgO-doped $LiNbO_3$ crystals (Oxide Corporation). A chromium layer of approximately 500 nm was sputtered onto the optically polished +*z*-face of the wafer. The optically polished –*z*-face was left uncoated. Subsequently, the wafer was cut into 5 × 15 mm² large chips by means of a wafer-dicing saw. The 15-mm-long edges of the chips were parallel to the crystal *x*-axis.

Poling and current measurement setup. The setup for calligraphic domain inversion and current measurement comprises two perpendicularly aligned high-precision linear stages (Aerotech ANT95-L) with a computerized numerical control. One stage is equipped with a temperature-controlled aluminium block to heat the crystal. The other stage carries a pivot-mounted cantilever to support the poling tip. A permanent magnet, mounted at the cantilever, and a static coil allow the adjustment of the contact force of the poling tip. Super-fine grain tungsten carbide rods (K-55SF) with 1 mm diameter serve as the starting material to prepare the poling tips. Taper grinding defines the tip geometry, and subsequent polishing with a diamond slurry ensures a tip radius of less than 10 µm (Extended Data Fig. 1).

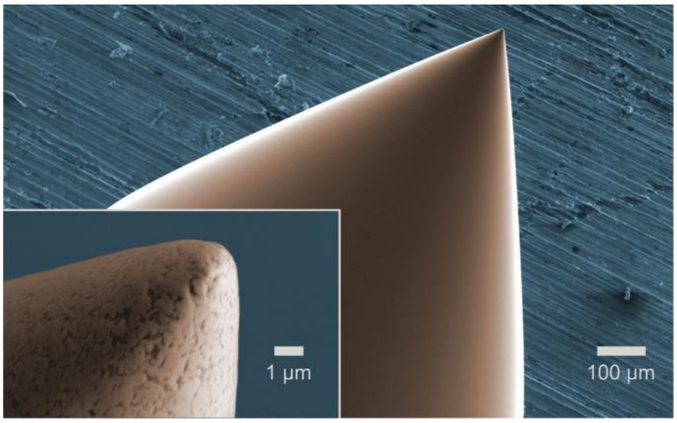

Extended Data Figure 1. Raster electron micrograph of a tungsten carbide tip prepared for calligraphic recording of charged domain walls in $LiNbO_3$.

For domain inversion, the poling tip is connected to a high-voltage source (Trek Model 10/10) while the crystal is glued to the aluminium block using silver conductive paste with the chromium coated side facing towards the aluminium. This ensures good electrical and thermal contact of the chip to the aluminium block. The tip approaches the crystal surface, achieves contact, and then moves along the crystal *x*-axis (Fig. 2b). Simultaneously, a homemade trans-impedance amplifier and a proportional-integral (PI) servo loop control the voltage at the poling tip to ensure a constant poling current of 30 nA. All domain lines were written at room temperature.

For measuring the conductive properties of the domain walls, the poling tip is connected to a high-voltage source with a lower maximum voltage but better signal-to-noise ratio (Trek Model 50/750). A commercial picoamperemeter (KeithleyPicoammeter 6485) measures the current through the domain wall at a given tip voltage. The tip can either move perpendicular across the previously written domain lines to obtain a spatial conductivity pattern or can be placed stationary right above a domain wall. Alternatively, conductive silver-paste, simultaneously connecting multiple domain lines at once, acts as a top electrode after domain inversion.

Piezoelectric force measurements (PFM) and conductive atomic force measurements (c-AFM). The measurements we carried out with a commercial atomic force microscope (NTEGRA from NT-MDT) equipped with a supplementary external lock-in amplifier (SR 830 from Stanford Research Systems) for the PFM measurements and a low noise current amplifier (DLPCA-200 from FEMTO Messtechnik GmbH) for the c-AFM measurements. The probes used were diamond coated (HA_HR_DCP from NT-MDT), exhibiting a tip radius of about 100 nm. For the PFM measurements, we applied an alternating voltage (f = some 10 kHz, U_{pp}=15 V) to the tip, and recorded the in-phase output channel of the lock-in amplifier. For the c-AFM measurements, we applied a DC voltage of 10 – 100 V to the Cr-bottom electrode, and the current was collected from the tip using the current amplifier. Typical scanning speed was set to few µm/s.
